# Supplementary material for: A nanobody-based molecular toolkit provides new mechanistic insight into clathrin-coat initiation
Source: eLife. 2019 Apr 30;8:e41768. doi: 10.7554/eLife.41768 (PMC6524969; doi:10.7554/eLife.41768)
Supplement: Transparent reporting form [file elife-41768-transrepform.pdf]

eLIFE
1st Floor
24 Hills Road
Cambridge CB2 1JP, UK
P 01223 855340
W elifesciences.org
T @elife

eLife's transparent reporting form

We encourage authors to provide detailed information *within their submission* to facilitate the interpretation and replication of experiments. Authors can upload supporting documentation to indicate the use of appropriate reporting guidelines for health-related research (see EQUATOR Network), life science research (see the BioSharing Information Resource), or the ARRIVE guidelines for reporting work involving animal research. Where applicable, authors should refer to any relevant reporting standards documents in this form.

If you have any questions, please consult our Journal Policies and/or contact us: editorial@elifesciences.org.

Sample-size estimation

- You should state whether an appropriate sample size was computed when the study was being designed
- You should state the statistical method of sample size computation and any required assumptions
- If no explicit power analysis was used, you should describe how you decided what sample (replicate) size (number) to use

Please outline where this information can be found within the submission (e.g., sections or figure legends), or explain why this information doesn't apply to your submission:

Computed sample-size estimation for the biochemical and cell-based experiments reported in this study is not relevant. The data presented are largely qualitative, and outcomes consistent both within and in between the results shown. Only small variations in the phenotypes and outcomes of the experimental attempts were observed. As the experiments build upon one another, reproducibility and correctness of the findings are perpetuated in the succession of experiments shown. For both the biochemical assays and cell-based experiments, repetitions were based on the well-accepted sample-size convention of three repeats. This allows clear evaluation of whether, and to what extent, any effect observed in the initial attempt is reproduced in the follow-up replicate experiments. For each experiment, the cell-based experiments were always conducted on duplicate coverslips, each containing thousands of adherent HeLa cells with a transfection efficiency of about 20-30%. This enables side-by-side comparison of identically processed untransfected and transfected cell groups in the same preparation. In the submission, this information is found in the figure legends and the Materials and Methods section.

Replicates

- You should report how often each experiment was performed
- You should include a definition of biological versus technical replication
- The data obtained should be provided and sufficient information should be provided to indicate the number of independent biological and/or technical replicates
- If you encountered any outliers, you should describe how these were handled
- Criteria for exclusion/inclusion of data should be clearly stated
- High-throughput sequence data should be uploaded before submission, with a private link for reviewers provided (these are available from both GEO and ArrayExpress)

Please outline where this information can be found within the submission (e.g., sections or figure legends), or explain why this information doesn't apply to your submission:

Since they were strictly preparative in nature, with defined and confirmable deliverables anticipated, the TALEN-based HeLa cell screen and the nanobody screen were performed only once. For the remainder of the investigation, experiments were conducted at least twice and routinely three times, as rationalized above. For this, the precise distinction between technical and biological replicates is spelled out at the beginning of the Materials and Methods section. For all the cell culture-based work, technical replicates in the form of duplicate coverslips were always included to permit systematic, but unblinded, comparison between the two replicates. As essentially all of the data presented were collected by only a single investigator, blinding was not possible. Selected but representative data is shown throughout, and no data were excluded.

Statistical reporting

- Statistical analysis methods should be described and justified
- Raw data should be presented in figures whenever informative to do so (typically when N per group is less than 10)
- For each experiment, you should identify the statistical tests used, exact values of N, definitions of center, methods of multiple test correction, and dispersion and precision measures (e.g., mean, median, SD, SEM, confidence intervals; and, for the major substantive results, a measure of effect size (e.g., Pearson's r, Cohen's d)
- Report exact p-values wherever possible alongside the summary statistics and 95% confidence intervals. These should be reported for all key questions and not only when the p-value is less than 0.05.

Please outline where this information can be found within the submission (e.g., sections or figure legends), or explain why this information doesn't apply to your submission:

As the visual experimental data and findings shown are largely qualitative in nature, no statistical methods were used to further analyze the results.

(For large datasets, or papers with a very large number of statistical tests, you may upload a single table file with tests, Ns, etc., with reference to sections in the manuscript.)

Group allocation

- Indicate how samples were allocated into experimental groups (in the case of clinical studies, please specify allocation to treatment method); if randomization was used, please also state if restricted randomization was applied
- Indicate if masking was used during group allocation, data collection and/or data analysis

Please outline where this information can be found within the submission (e.g., sections or figure legends), or explain why this information doesn't apply to your submission:

Group allocation is not germane to this study. No human or animal participants that were compared to one another were used.

Additional data files ("source data")

- We encourage you to upload relevant additional data files, such as numerical data that are represented as a graph in a figure, or as a summary table
- Where provided, these should be in the most useful format, and they can be uploaded as "Source data" files linked to a main figure or table
- Include model definition files including the full list of parameters used
- Include code used for data analysis (e.g., R, MatLab)
- Avoid stating that data files are "available upon request"

Please indicate the figures or tables for which source data files have been provided:

Not applicable.

eLife Sciences Publications, Ltd is a limited liability non-profit non-stock corporation incorporated in the State of Delaware, USA, with company number 5030732, and is registered in the UK with company number FC030576 and branch number BR015634 at the address 1st Floor, 24 Hills Road, Cambridge CB2 1JP | August 2014
